# Archaeal Communities in a Heterogeneous Hypersaline-Alkaline Soil

**DOI:** 10.1155/2015/646820

**Published:** 2015-05-13

**Authors:** Yendi E. Navarro-Noya, César Valenzuela-Encinas, Alonso Sandoval-Yuriar, Norma G. Jiménez-Bueno, Rodolfo Marsch, Luc Dendooven

**Affiliations:** ^1^Cátedras CONACYT, Centro de Investigación en Ciencias Biológicas, Universidad Autónoma de Tlaxcala, Km. 10.5 Autopista Tlaxcala-Texmelucan, 90120 San Felipe Ixtacuixtla, TLAX, Mexico; ^2^Laboratory of Soil Ecology, ABACUS, Cinvestav, Avenida Instituto Politécnico Nacional 2508, 07360 Mexico City, DF, Mexico

## Abstract

In this study the archaeal communities in extreme saline-alkaline soils of the former lake Texcoco, Mexico, with electrolytic conductivities (EC) ranging from 0.7 to 157.2 dS/m and pH from 8.5 to 10.5 were explored. Archaeal communities in the 0.7 dS/m pH 8.5 soil had the lowest alpha diversity values and were dominated by a limited number of phylotypes belonging to the mesophilic Candidatus *Nitrososphaera*. Diversity and species richness were higher in the soils with EC between 9.0 and 157.2 dS/m. The majority of OTUs detected in the hypersaline soil were members of the Halobacteriaceae family. Novel phylogenetic branches in the Halobacteriales class were detected in the soil, and more abundantly in soil with the higher pH (10.5), indicating that unknown and uncharacterized Archaea can be found in this soil. Thirteen different genera of the Halobacteriaceae family were identified and were distributed differently between the soils. *Halobiforma*, *Halostagnicola*, *Haloterrigena*, and *Natronomonas* were found in all soil samples. Methanogenic archaea were found only in soil with pH between 10.0 and 10.3. Retrieved methanogenic archaea belonged to the Methanosarcinales and Methanomicrobiales orders. The comparison of the archaeal community structures considering phylogenetic information (UniFrac distances) clearly clustered the communities by pH.

## 1. Introduction

Hypersaline environments are found in natural or anthropogenic aquatic or terrestrial habitats in most parts of the world [1]. Athalassohaline and thalassohaline aquatic systems, saline deserts, solonchaks, solar salterns, and former lakebeds are examples of them. Cultivation- and molecular-based studies have been used to reveal the diversity of bacterial and archaeal communities in hypersaline environments [2–6]. In such hypersaline habitats, the archaeal community dominates the microbial population [1, 7]. The majority of the Archaea identified in such environments belong to the Halobacteriales [7].

Soda lakes are exceptional hypersaline (up to saturation) aquatic habitats that have simultaneously an extreme alkaline pH. Several soda lakes from around the world have been studied [8–12]. Novel Archaea have been isolated from these soda lakes bettering our understanding of the physiology, ecology, and distribution of polyextremophiles, such as the haloalkaliphiles [13]. Their terrestrial counterparts, for example, soda desert, however, remain largely unexplored and we still have an inadequate understanding of terrestrial Archaea [7, 14]. Previous studies on Archaea in hypersaline soil focused often on one salinity level [1, 13, 15]. Consequently, our understanding of how a salinity gradient affects archaeal diversity and their functionality is limited.

The soil of the former lake Texcoco (Mexico) is a unique extreme-haloalkaline terrestrial ecosystem formed from volcanic ash deposited* in situ* in a lacustrine environment covered recently by colluvial materials. The lake Texcoco covered 50% of the original lakes around the Aztec city of Tenochtitlan (current day Mexico City). The lakes were drained since the 17th century, and, nowadays, it is a large area of lacustrine bed exposed to desertification [16]. Since the beginning of the 1970s, a hydraulic drainage system has been installed and the soil irrigated with wastewater effluent to reduce the salt content and pH so that the former lakebed can be vegetated. The artificial drainage together with the variability inherent of the former lakebed has generated a heterogenic soil [16]. According to the FAO soil classification [17], a very strong saline soil has an electrolytic conductivity (EC) > 16 dS/m. Soils with EC 16.3 dS/m have been described in other studies as “extremely saline soils” [18]; the EC in soil of Texcoco can reach 100 dS/m and the pH can be as high as 10.5 [16]. Culture-based studies with soil of former lake Texcoco have yielded a number of novel prokaryotic species, that is, the Archaea* Natronobacterium texcoconense* ^T^ and* Natronorubrum texcoconense* ^T^ and the Bacteria* Texcoconibacillus texcoconensis* ^T^ [19–21]. Valenzuela-Encinas et al. [22–24] first studied the bacterial and archaeal communities using cloned sequences of the 16S rRNA gene. It was found that in the soil with EC 159 dS/m and pH 10.5 more than 95% of the clones were affiliated with members of the family Halobacteriaceae belonging to phylum Euryarchaeota [22], while in the drained soil with EC 0.68 dS/m and pH 7.8 most of the cloned Archaea were related to mesophilic Crenarchaeota and were not-yet-cultured [24]. Few studies described the microbial community in the remaining lake [25, 26].

It is most likely that the soil of the former lake Texcoco harbors more novel archaeal species with unique characteristics. However, the extent to which new archaeal species can be found in this environment has not been determined. In this study, archaeal-specific primers combined with taxon-based and phylogenetic approaches were used to investigate and identify archaeal diversity patterns in soil from the former lake Texcoco with different EC (0.7–157.2 dS/m) and pH (8.5–10.5).

## 2. Material and Methods

### 2.1. Site Description and Soil Sampling

The sampling site is located in the former lake Texcoco (northern latitude 19°29′46′′, western longitude 98°58′01′′) in the State of Mexico, Mexico, at an altitude of 2240 m.a.s.l., with a mean annual temperature of 16°C and mean annual precipitation of 705 mm. Soil was sampled from six different locations with different EC and pH. At each location, the 0–10 cm soil layer was sampled five times from three 20 m^2^ areas with a small hand spade in August 2013. The soil collected from each area was pooled separately so that eighteen soil samples were obtained. The soil samples were taken to the laboratory in a black polyethylene bag kept on ice. The eighteen samples were 5 mm sieved separately under aseptic conditions. The soil was characterized and a 25 g subsample was stored at −20°C for less than three weeks until extracted for DNA.

Soil pH was measured in 1 : 2.5 soil-H_2_O suspension using a glass electrode [27]. The EC was measured according to the saturated paste method [28]. The total carbon (TOC) in soil was determined by oxidation with K_2_Cr_2_O_7_ and trapping the evolved CO_2_ in NaOH, followed by titration with 0.1 M HCl [29]. Total nitrogen (TN) was measured by the Kjeldahl method [30] and soil particle size distribution by the hydrometer method as described by Gee and Bauder [31]. The water holding capacity (WHC) was measured on soil samples water-saturated in a funnel and left to stand overnight (Table 1).

### 2.2. DNA Isolation and PCR-Amplification of Archaeal 16S rRNA Genes

Metagenomic DNA was extracted from soil samples using the Power Soil DNA Isolation Kit (MO BIO Laboratories, CA, USA) following the manufacturer's instructions. The V1–V3 region (about 550 bp) of archaeal 16S rRNA gene was chosen for amplification and subsequent pyrosequencing. The DNA samples were amplified using the set of archaeal primers 25F 5′-CYG GTT GAT CCT GCC RG-3′ [32] and A571R 5′-GCT ACG GNY SCT TTA RGC-3′ [33]. Each ribosomal primer set was flanked by a 454-adapter sequence. A 10-nucleotide tag was incorporated between the 454-adapter and the forward primer for sample identification among mixed amplicon libraries. PCR products per soil sample were amplified in triplicate with a 30-cycle-based protocol, pooled, and purified using the DNA Clean and Concentrator Columns (Zymo Research, Irvine, CA, USA). Each library was quantified using NanoDrop 2000 (Thermo Fisher Scientific Inc., Suwanee, GA, USA) and mixed in equal amount. Sequencing was done unidirectionally by Macrogen Inc. (Seoul, Korea) using the Roche 454 GS-FLX Titanium (Roche 454 Life Sciences, Branford, CT, USA).

### 2.3. Pyrosequencing Reads Processing

Sequences were processed for quality, barcode sorting, and denoising through the QIIME pyrosequencing pipeline (http://qiime.org/). Briefly, reads shorter than 250 nt, with quality scores less than 25, or containing errors in adaptors and primers were discarded. One mismatch was allowed in the barcode sequence. Denoising of the reads was done with the script* denoise_wrapper.py* using the barcode-sorted libraries and the standard flowgram format (SFF) files as inputs [34]. Sequences are available at the Sequence Read Archive (SRA) under the accession number SRP041362.

The screened sequences were used to determine* de novo* operational taxonomic units (OTUs) at 97% cut-off with the script* pick_de_novo_otus.py*. One representative sequence for each OTU was chosen, and potentially chimeric sequences were detected using ChimeraSlayer [35] and removed from the representative sequences dataset.

### 2.4. Taxon-Based and Phylogenetic Analyses

The taxonomic assignments were done with the naïve Bayesian rRNA classifier from the Ribosomal Data Project (http://rdp.cme.msu.edu/classifier/classifier.jsp) and the Greengenes reference database at a confidence threshold of 80% [36]. The obtained biological observation matrix (BIOM) table was rarefied to 1,200 reads to avoid bias in diversity analysis by differences in sampling-sequencing effort (Figure S1) (see Supplementary Material available online at http://dx.doi.org/10.1155/2015/646820). Diversity (Shannon, Simpson, and Phylogenetic diversity indices) and species richness estimator Chao1 were calculated using the rarified datasets within QIIME pipeline using the script alpha_rarefaction.py. The relative abundance was calculated for OTU and genus-taxonomic level in each sample. Variables in the tables of occurrence with no normal distribution were log transformed.

A network plot representing the presence of the OTUs in the soil samples was done. OTUs and samples are designated as two types of nodes in a bipartite graph in which OTU-nodes are connected via edges to sample-nodes. Edge weights are defined as the number of sequences in a given OTU. To cluster the OTUs and samples in the network, a stochastic spring-embedded algorithm implemented in Cytoscape version 3.0.2 was used [37].

The representative sequence dataset was aligned at a minimum percent sequence identity of 75% using PyNast [38]. Sequences that could not be aligned were removed. Bootstrapped neighbor joining phylogenetic trees were constructed with evolutionary distances obtained by a Maximum Likelihood approach within the QIIME pipeline [39]. Phylogenetic information was also used to calculate pairwise UniFrac distance matrices using weighted data within QIIME. Cluster analyses were done using the UniFrac pairwise distance matrix using Unweighted Pair Group Method with Arithmetic Mean (UPGMA). Robustness determination of individual UPGMA clusters was performed by comparing rarefied UPGMA trees to either (full or consensus) tree for jackknife support of tree nodes. Canonical Correspondence Analysis (CCA) was used to summarize overall relationships among environmental variables and the observed species assemblages. The CCA was run in R (*vegan* package (http://cran.r-project.org/web/packages/vegan/index.html)).

## 3. Results

**Figure 1:**
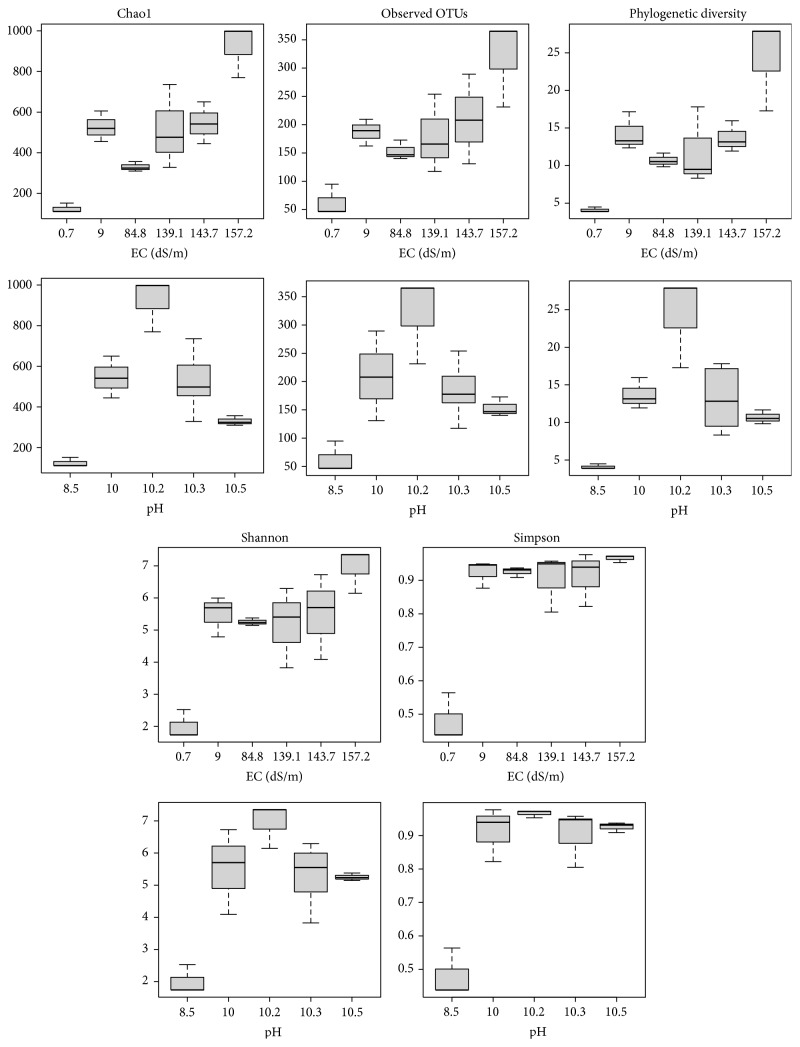
Boxplots of the alpha diversity parameters of the archaeal communities in alkaline-saline soils of the former lake Texcoco with different electrolytic conductivity and pH.

The sequencing of eighteen libraries yielded 75,727 V1–V3 archaeal 16S rRNA raw sequence reads, with an average length of 486 nt. After quality filtering, denoising, and chimera detection, a total of 1477* de novo* OTUs (3% cut-off) were found. Five different parameters of alpha diversity, that is, Chao1 richness estimator, observed OTUs, phylogenetic diversity, and Shannon and Simpson indices, exhibited different patterns in terms of diversity and species richness depending on the EC or pH values in extremely saline and alkaline soils (Figure 1). The lowest diversity was found in the soil with the least extreme environmental conditions, that is, EC 0.7 dS/m and pH 8.5. The highest values for species richness, phylogenetic diversity, and heterogeneity were found in soil with EC 157.2 dS/m and pH 10.2. The archaeal communities in the soil with higher salinity and alkalinity had a similar evenness as determined by the Simpson index.

**Figure 2:**
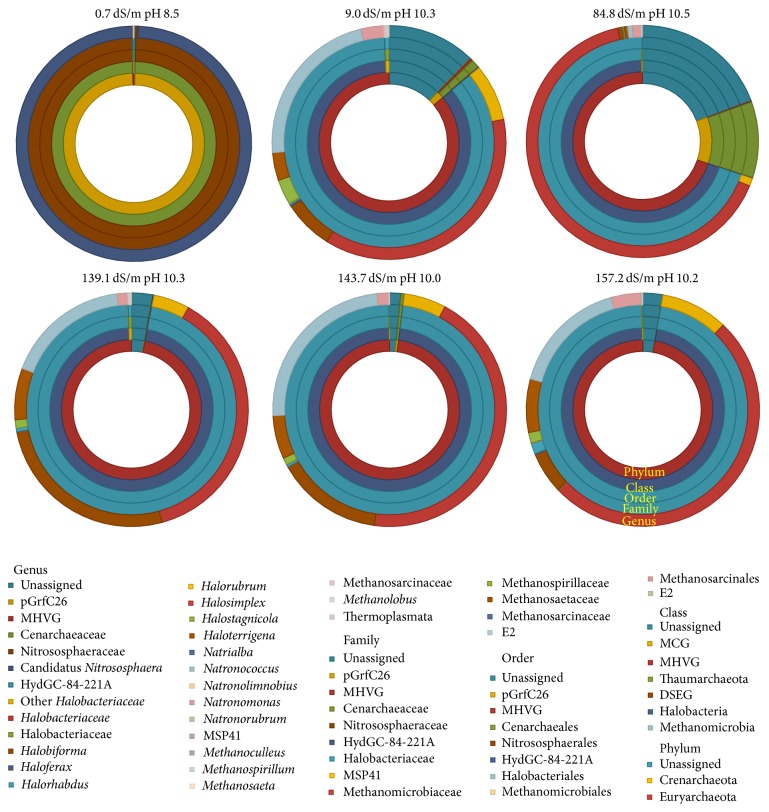
Taxonomic distribution at different ranks of the archaeal communities in alkaline-saline soils of the former lake Texcoco. Soils are different in electrolytic conductivity and pH.

A summary of the taxonomic distribution of the soil archaeal communities at different taxonomic ranks can be found in Figure 2. The taxonomic assemblages showed important differences. While more than 99% of the OTUs in the 0.7 dS/m and pH 8.5 soil belonged to the Crenarchaeota phylum, only between 0.005 and 10% were found in the other soils. The Euryarchaeota phylum dominated in the soils with higher salinity and alkalinity (69.8–97.9% of all OTUs). Within the Euryarchaeota phylum, Halobacteria, Methanomicrobia, and Thermoplasmata were identified, with the Halobacteria as the most abundant (69.7–97.8%).

At lower taxonomic levels, nine orders, 12 families, and 28 genera were identified. A more detailed summary of the genus distribution can be found in Table 2. The largest relative abundance of Crenarchaeota was found in soil with EC 0.7 dS/m and pH 8.5 (99%) and soil with EC 84.8 dS/m and pH 10.5 (10%). However, different groups dominated them. Candidatus* Nitrososphaera* dominated in soil with EC 0.7 dS/m and pH 8.5 and members of the Cenarchaeaceae family in the 84.8 dS/m and pH 10.5 soil. Only some of the OTUs belonging to the Halobacteriaceae family could be assigned to genus levels and the archaeal communities had different distributions considering these genera (Table 2). Thirteen different Halobacteriaceae genera were detected in the soils. The genera with more than 0.5% of the OTUs were* Halobiforma*,* Halorhabdus, Halostagnicola, Haloterrigena, Natronococcus, *and* Natronomonas.* Methanogenic Archaea were found only in soil with pH values between 10.0 and 10.3.

**Figure 3:**
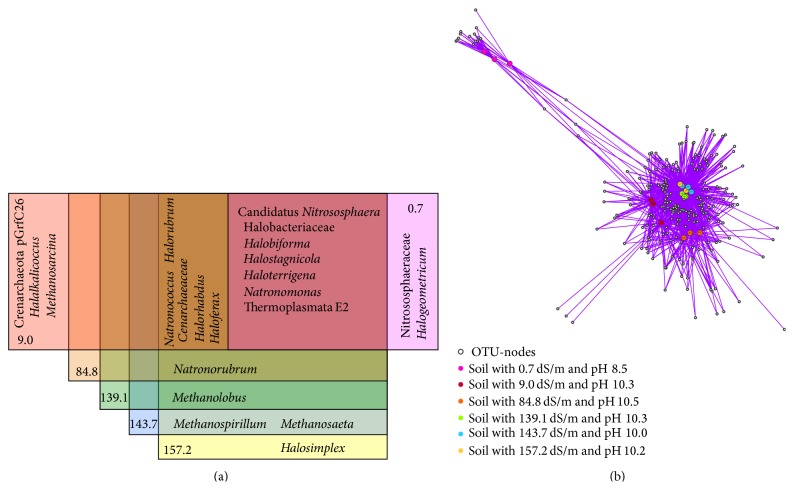
Shared taxonomic groups and operational taxonomic units (OTUs) at 97% cut-off in alkaline-saline soils of the former lake Texcoco with different electrolytic conductivity and pH. (a) Venn diagrams of cooccurrence of taxonomic groups. (b) Network plot of occurrence of OTUs. OTU-nodes (white circles) are connected via edges (lines) to sample-nodes (large circles) in which their sequences are found. Edge weights (lines' length) are defined as the number of sequences in an OTU.

Many of the detected genera were found in all soil samples, that is, Candidatus* Nitrososphaera, Halobiforma, Halostagnicola, Haloterrigena, Natronomonas,* and members of Thermoplasmata.* Natronococcus*,* Halorhabdus, Haloferax,* and members of the family Cenarchaeaceae were found in soil with EC > 9.0 dS/m (Figure 3(a)). The genera detected in soil with EC > 84.8 dS/m were* Natronorubrum*, EC > 139.1 dS/m* Methanolobus*, EC > 143.7 dS/m* Methanospirillum* and* Methanosaeta*, and* Halosimplex* detected uniquely in soil with EC 157.2 dS/m. At the OTU level, the network analysis also revealed a low number of OTUs unique to a given EC (Figure 3(b)). In this analysis the length of the edges is weighted by the abundance of the OTUs. The soil with EC 0.7 dS/m and pH 8.5, as well as EC 9.0 dS/m and pH 10.0, harbors OTUs with a high relative abundance not shared with the other soil samples. OTUs of* Halosimplex*, found uniquely in the soil with EC 157.2 dS/m, were not highly evident in the network analysis because of their low abundance.

**Figure 4:**
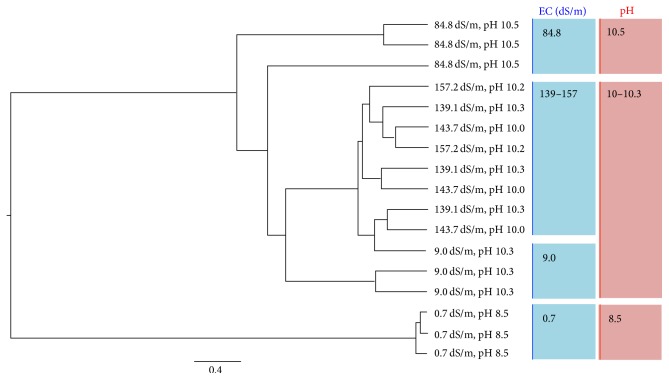
UPGMA jackknifed clustering of the UniFrac weighted distances for rarefied 1200-sequence reads (*n* = 17) of the archaeal communities from alkaline-saline soils of the former lake Texcoco with different electrolytic conductivity and pH. The scale bar represents 0.4% of divergence UniFrac over all sites analyzed.

**Figure 5:**
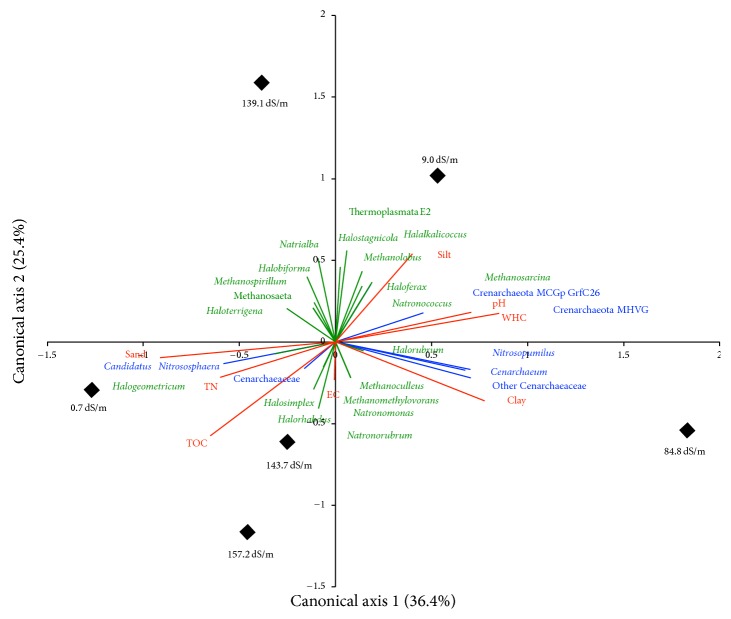
Canonical correlation analysis considering the percentages of the taxonomic groups and eight soil characteristics (particle size distribution (CLAY, SAND, and SILT), pH, electrolytic conductivity (EC), water holding capacity (WHC), total nitrogen (TN), and total organic carbon (TOC)) in alkaline-saline soils of the former lake Texcoco with different electrolytic conductivity and pH. Blue taxonomic groups are Crenarchaeota members; green taxonomic groups are Euryarchaeota members.

Comparing the archaeal communities considering the OTUs distribution combined with phylogenetic information (Figure 4), the jackknifed cluster analysis identified three groups of archaeal communities: (i) soil with EC 0.7 dS/m and pH 8.5; (ii) soil with 9.03–157.2 dS/m and pH 10–10.3; and (iii) soil with EC 84.8 dS/m and pH 10.5. The canonical correlation analysis considering soil characteristics (Figure 5) revealed also three groups: (i) soil with EC 0.7, 143, and 157.2 dS/m; (ii) soil with EC 9.03 and 139.1 dS/m; and (iii) soil with EC 84.8 dS/m.

## 4. Discussion

In this study, the archaeal diversity was investigated in six soils from the same area, but with different EC and pH (0.7–157.2 dS/m and 8.5–10.5). Taxon-based and phylogenetic analyses revealed that the archaeal community in soil with EC 0.7 dS/m and pH 8.5 resembled those found in neutral and nonsaline soils, that is, two or three dominant phylotypes belonging to the mesophilic Crenarchaeota [2–4, 40]. Diversity and species richness were lowest in this soil. Remarkably, archaeal communities from soils with extremely higher values of EC and pH exhibited the largest alpha diversity. It has been reported that environments with greater temporal fluctuations in salinity showed a larger archaeal diversity and species richness [7]. This phenomenon is also observed in terrestrial ecosystem as the soil of the former Lake Texcoco, which is not changing but heterogenic.

Phylogenetic analyses revealed that the dominating OTUs in soil with EC 0.7 dS/m and pH 8.5 were assigned as Candidatus* Nitrososphaera* belonging to the Crenarchaeotal group 1.1b or soil Crenarchaeotal group, a deeply divergent clade distantly related to hyperthermophiles [40], and from the recently suggested Thaumarchaeota phylum [41]. Crenarchaeota from the 1.1a and 1.1b groups are thought to play an important role in the nitrogen cycle in soil and planktonic marine systems as ammonium oxidizers [42].

As was expected, the majority of the OTUs detected in the highly haloalkaline soils were members of the Halobacteriaceae family (belonging to the monophyletic class Halobacteria). Halobacteriales dominate organic matter degradation in hypersaline environments [43]. Pure isolates of halophilic Archaea, belonging to the class Halobacteria, include currently 38 genera [7], and, considering that the haloalkaliphilic Archaea are physiologically distinct Haloarchaea, 13 genera were detected in this study. Taxonomic assignation revealed that the most abundant genera in soils of the former lake Texcoco were* Natronococcus* and* Natronomonas. Natronococcus *genus was one of the first described haloalkaliphilic Archaea [44] and comprises moderate halophilic species with a growth range between 1.5 and 5 [Na^+^] M, 6.5–10 pH, and 22–50°C [45].

Venn diagrams revealed that* Halobiforma, Halostagnicola, Haloterrigena,* and* Natronomonas* were found in all soil samples. This indicates two possibilities: (i) these genera possess the capacity to survive in soils with highly variable salt contents (ii) and/or possess a great dispersal capacity. Likewise, unique diversity was found, being more abundant in the soil with the lowest (0.7 dS/m) EC and 9.0 dS/m, indicating archaeal populations adapted to the specific conditions of each soil. Haloalkaliphilic adaptations require modifications of the intracellular components, that is, specialized protein amino acid compositions to maintain solubility, structural flexibility, and water availability necessary for enzyme function [1, 46, 47]. These specific adaptations narrowed the ability of some Archaea to grow in different environmental conditions. Certainly, the shared and unique genera identified in this study contain haloalkaliphilic species that are known to grow strictly in haloalkaline conditions. However, 36.6% to 63.4% of the OTUs found in soil with EC 9.0–157.2 dS/m were assigned as Halobacteriaceae members but could not be assigned to the genus level. A phylogenetic analysis (Figure S2) placed the OTUs between the cultured* Halobacteria* species or they represented deep phylogenetic branches within the Halobacteriales. This indicates that a largely unexplored archaeal population existed in the heterogeneous extreme saline-alkaline soil. The same results have been reported in other ecological studies of Archaea where the retrieved OTUs did not have close relatives in public databases [7, 48]. It is possible that the databases are still biased to the few dominant species with widespread geographical distribution and widely reported [15, 49, 50]. Metagenomic analyses frequently rely on the assumption that undiscovered microorganisms will have a degree of similarity to those already known, creating a potential bias against novel phylotypes. There is still a need for community genomics and* de novo* sequence assembly to determine the biological diversity in extreme environments as used in this study, that is, a soil with EC 157.2 dS/m and pH 10.5. The discovery of novel microorganisms is a major incentive driving metagenomic investigations in many habitats worldwide. The soil of the former lake Texcoco is a promising and an exceptional ecosystem. It has the potential to yield new genes and species and might be a source of new biomolecules.

Retrieved sequences belonging to methanogenic Archaea were identified as* Methanolobus, Methanosaeta, Methanomethylovorans* and* Methanosarcina* (Methanosarcinales), and* Methanoculleus* and* Methanospirillum* (Methanomicrobiales). The methanogenic Archaea isolated or detected through molecular approaches (16S rRNA and/or* mcr*A gene sequence analysis) from haloalkaline environments include the genera, for example,* Methanobacterium, Methanocalculus, Methanoculleus, Methanolobus, Methanosalsum,* and* Methanosarcina* [1, 51–54]. Pure cultures of some alkaliphilic methanogenic Archaea are able to grow up to pH 10.2 [55], while in sediment of soda lakes methanogenic activity was found within the alkaline pH range between 8 and 10.5 [56]. In general, the methanogenic Archaea distribution in this study was more delimited as they were detected only in soil with pH 10.0–10.3 and* Methanosarcina* only in soil with EC 9.0 dS/m and pH 10. Methanogenic Archaea are of great biotechnological interest due to their use in wastewater treatment and, recently, biogas production [11]. However, their strictly anaerobic physiology has restricted their isolation at haloalkaline environments such as soda lake sediments. Inoculum for reactors of methane production from soda lake sediments showed lower yields using acetate or hydrogen as substrates because methanogens compete for the electron donors with other anaerobes, such as sulfate and sulfur reducers, also present in the inoculum [11, 57]. The haloalkaliphilic archaeal communities in soil of the former lake Texcoco might be a good source to isolate these Archaea with this biotechnological purpose.

Species richness of the archaeal communities was similar for different Texcoco soils. However, their abundance was highly different. The comparison of the archaeal community structures considering phylogenetic information (UniFrac distances) clustered the communities by pH rather than EC. It was previously suggested that environments with regular salinity fluctuations might allow the coexistence of archaeal members with a wide range of salt-tolerance, that is, halotolerant, halophilic, and nonhalophilic [7]. Their distribution might be determined by other soil characteristics, for example, pH and/or WHC. When considering the taxonomic distributions and soil characteristics, Crenarchaeota and Thermoplasmata, as well as size particle distribution, separated the archaeal communities. It was believed, until recently, that archaeal ecology was restricted to extreme environments. However, new molecular tools have been revealing the hidden, wide, and ubiquitous diversity of the Archaea domain. It was hypothesized that “archaeal communities were more similar within habitats than among habitats” when comparing broad environmental gradients and habitat types [2]. The archaeal domain is certainly ubiquitous, but Archaea have developed very specialized functions depending on the physical and chemical characteristics of their environments. In the same terrestrial area, but with different values of pH and EC, it is highly probable that archaeal populations participate in completely different biogeochemical processes. While in nonsaline soils well recognized ammonium oxidizers were detected (clade 1.1b and Thaumarchaeota), in saline and hypersaline soils Archaea with organic matter recycling capabilities were found (Halobacteriales).

## 5. Conclusion

Phylogenetic and taxon-based analyses revealed the following: (i) Archaeal diversity and species richness in the soil with EC ranging from 9.0 to 157.2 dS/m were higher than in soil with EC 0.7 dS/m. (ii) The identified Halobacteriales genera have, generally, haloalkaliphilic representatives (*Halalkalicoccus, Halobiforma, Halorubrum, Halostagnicola, Haloterrigena, Natrialba, Natronococcus, Natronomonas, and Natronorubrum*). (iii) Novel phylogenetic branches in the Halobacteriales class were found in the soil with EC 9.0–157.2 dS/m indicating that unknown and uncharacterized Archaea can be found in these poorly characterized hyperhaloalkaline soils. (iv) OTUs related with methanogenic Archaea were found only in soil with pH 10.0–10.3. (v) Most of the OTUs were ubiquitous, but their distribution was different. (vi) Archaeal community structures considering phylogenetic information were correlated with pH. (vii) The archaeal populations were well defined by soil conditions.

## Supplementary Material

Supplementary Figure 1: Rarefaction curves were constructed with the observed OTUs to verify the sampling–sequencing effort (Fig. S1). The observed species richness is larger in soil with electrolytic conductivity (EC) 157.2 dS/m, and lower in soil with EC 0.7 dS/m.
Supplementary Figure 2: A phylogenetic tree was constructed with the sequences found in this study so as to have a more detailed phylogenetic placement of the archaeal phylotypes (Fig. S2). In the Crenarchaeota phylum, the mesophilic Crenarchaeota (ammonia oxidation clade) grouped the majority of the OTUs from the 0.7 dS/m soil. A group of abundant OTUs in this soil was closely affiliated with Candidatus *Nitrososphaera gargensis* recently suggested as the phylum Thaumarchaeota. OTU 144 with a high abundance in the 0.7 dS/m and 9.0 dS/m soils represented a deep branch in the group 1.1b or soil Crenarchaeota.
Some OTUs belonging to Halobacteriales were detected in both the 9.0 and 139.1 dS/m soils. In general, the OTUs that were more abundant in the MEDIUM soil were more closely related with known genera and species, for example, *Halosarcina palida, Halorhabdus tiamatea, Natronococcus amylolyticus*, while OTUs more abundant in 157.2 dS/m soil clustered in different mostly unknown branches.

## Figures and Tables

**Table 1:** Some physical-chemical characteristics of soils from the former lake Texcoco.

Soil sampling site	EC^a^ (dS/m)	pH	WHC^b^	Total N	Total C	Particle size distribution		
						Clay	Silt	Sand
			(g/kg soil)					
TX_1	0.7	8.5	851	1.9	32.8	127	247	623
TX_2	9.0	10.3	1046	1.3	23.2	270	341	181
TX_3	84.8	10.5	1120	0.9	16.7	653	270	77
TX_4	139.1	10.3	850	0.9	22.6	332	216	462
TX_5	143.7	10.0	923	0.9	30.9	332	92	576
TX_6	157.2	10.2	747	1.4	46.7	478	131	391

^a^EC: electrolytic conductivity; ^b^WHC: water holding capacity.

**Table 2:** Relative abundance of the taxonomic affiliations at genus level of the archaeal communities of soils from the former lake Texcoco.

Taxonomic group	Electrolytic conductivity (dS/m)					
	0.7	9.0	84.8	139.1	143.7	157.2
Candidatus *Nitrososphaera *	99.09 (0.10)^a^	0.13 (0.09)	0.08 (0.02)	0.14 (0.05)	0.04 (0.02)	0.01 (0.01)
Cenarchaeaceae^b^	0	1.06 (0.60)	9.10 (4.48)	0.02 (0.01)	0.29 (0.14)	0.01 (0.01)
*Cenarchaeum *	0	0.01 (0.01)	0.05 (0.02)	0	0.01 (0.01)	0
Crenarchaeota MCGp GrfC26	0	0.08 (0.04)	0	0	0	0
Crenarchaeota MHVG	0	0.26 (0.18)	0.16 (0.12)	0	0.01 (0.01)	0
*Halalkalicoccus *	0	0.01 (0.00)	0	0	0	0
Halobacteriaceae^b^	0.09 (0.04)	36.83 (4.79)	63.40 (10.57)	40.97 (10.54)	46.16 (11.30)	48.42 (1.66)
Halobacteriaceae XKL75	0	0.01 (0.01)	0.01 (0.01)	0.02 (0.02)	0	0.02 (0.02)
Halobacteriales MSP41	0	0	0	0.07 (0.04)	0.01 (0.01)	0.16 (0.06)
*Halobiforma *	0.01 (0.01)	3.17 (1.56)	0.43 (0.04)	8.39 (3.50)	6.23 (2.82)	2.58 (0.17)
*Haloferax *	0	0.18 (0.09)	0.01 (0.01)	0.02 (0.02)	0.03 (0.02)	0.03 (0.02)
*Halogeometricum *	0.01 (0.01)	0	0	0	0	0
*Halorhabdus *	0	0.40 (0.04)	0.43 (0.07)	0.51 (0.30)	0.83 (0.56)	2.44 (0.85)
*Halorubrum *	0	0.03 (0.03)	0.01 (0.01)	0	0.03 (0.02)	0.01 (0.01)
*Halosimplex *	0	0	0	0	0	0.01 (0.00)
*Halostagnicola *	0.02 (0.01)	5.41 (2.19)	0.13 (0.06)	3.14 (0.86)	2.90 (1.53)	1.73 (0.17)
*Haloterrigena *	0.01 (0.01)	4.11 (1.48)	0.40 (0.05)	9.77 (2.26)	6.72 (1.11)	8.68 (2.70)
*Methanoculleus *	0	0	0	0	0.01 (0.01)	0
*Methanolobus *	0	0.06 (0.04)	0	0.01 (0.01)	0	0
*Methanomethylovorans *	0	0	0	0	0.01 (0.01)	0
*Methanosaeta *	0	0	0	0.01 (0.01)	0.01 (0.01)	0
*Methanosarcina *	0	0.01 (0.01)	0	0	0	0
*Methanospirillum *	0	0	0	0.02 (0.02)	0.01 (0.01)	0
*Natrialba *	0	0.01 (0.01)	0	0.04 (0.03)	0	0.01 (0.01)
*Natronococcus *	0	19.90 (4.57)	0.68 (0.26)	16.38 (5.58)	20.40 (8.13)	15.87 (0.26)
*Natronomonas *	0.01 (0.01)	3.32 (0.80)	1.29 (0.16)	1.23 (0.52)	2.00 (0.67)	4.96 (1.00)
*Natronorubrum *	0	0	0.01 (0.01)	0	0	0.10 (0.10)
*Nitrosopumilus *	0	0.20 (0.13)	1.80 (0.86)	0	0.06 (0.04)	0
Other Cenarchaeaceae^c^	0	0.01 (0.01)	0.18 (0.07)	0	0.03 (0.03)	0
Other Halobacteriaceae^c^	0.01 (0.01)	11.37 (1.08)	1.28 (0.23)	15.94 (3.20)	11.76 (1.29)	11.27 (0.53)
Thermoplasmata E2	0.08 (0.05)	0.59 (0.30)	0.13 (0.09)	0.54 (0.08)	0.14 (0.12)	0.26 (0.16)

^a^Numbers in parenthesis are standard errors.

^b^OTUs with not enough sequence information to reach a deeper taxonomic assignation.

^c^Novel groups not classified yet.
