# Supplementary figures and images for: Archaeal Communities in a Heterogeneous Hypersaline-Alkaline Soil
Source: Archaea. 2015 May 13;2015:646820. doi: 10.1155/2015/646820 (PMC4444560; doi:10.1155/2015/646820)

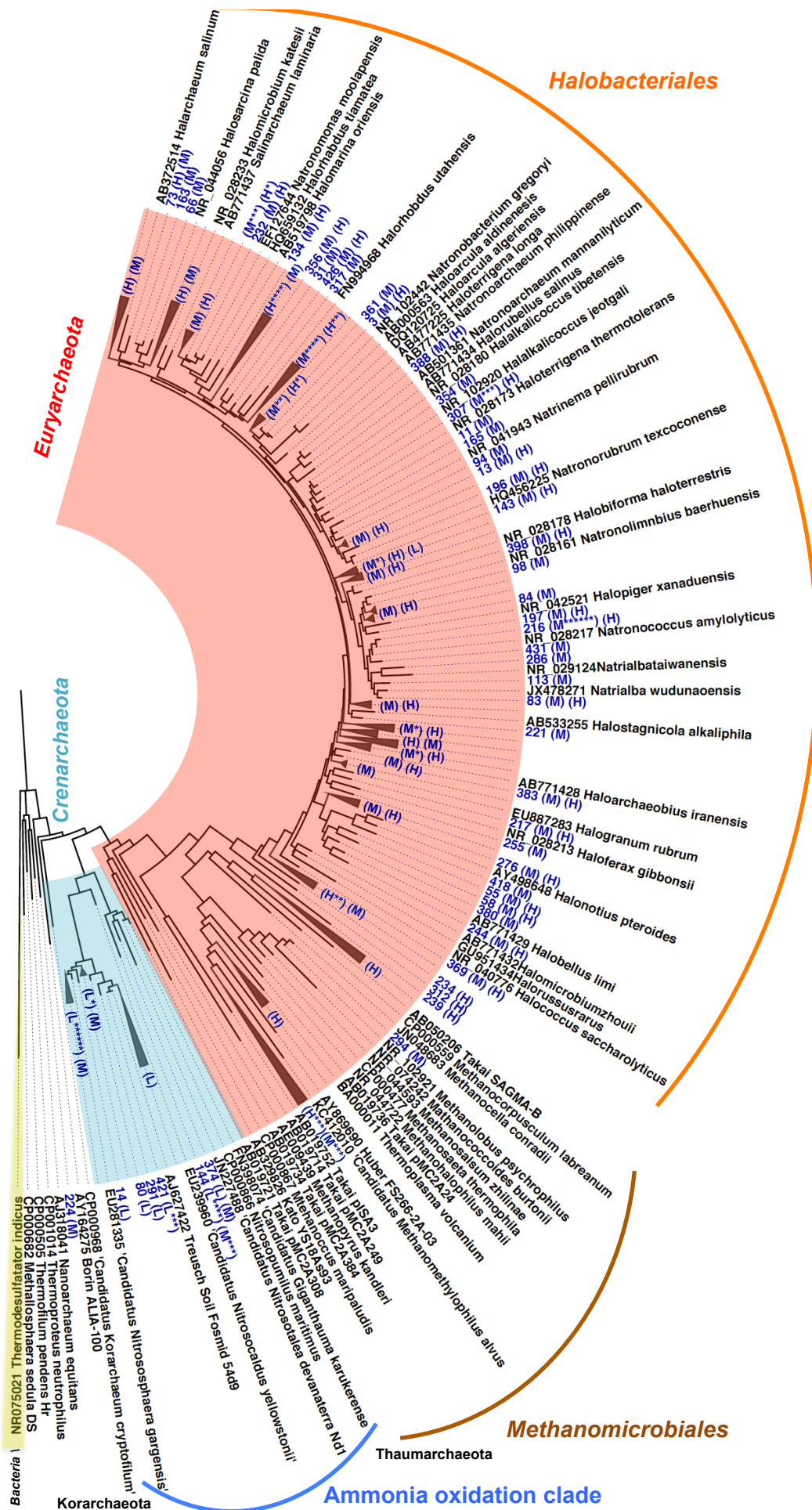

Supplement: Supplementary file 1 — Supplementary Figure 1: Rarefaction curves were constructed with the observed OTUs to verify the sampling–sequencing effort (Fig. S1). The observed species richness is larger in soil with electrolytic conductivity (EC) 157.2 dS/m, and lower in soil with EC 0.7 dS/m. Supplementary Figure 2: A phylogenetic tree was constructed with the sequences found in this study so as to have a more detailed phylogenetic placement of the archaeal phylotypes (Fig. S2). In the Crenarchaeota phylum, the mesophilic Crenarchaeota (ammonia oxidation clade) grouped the majority of the OTUs from the 0.7 dS/m soil. A group of abundant OTUs in this soil was closely affiliated with Candidatus Nitrososphaera gargensis recently suggested as the phylum Thaumarchaeota. OTU 144 with a high abundance in the 0.7 dS/m and 9.0 dS/m soils represented a deep branch in the group 1.1b or soil Crenarchaeota. Some OTUs belonging to Halobacteriales were detected in both the 9.0 and 139.1 dS/m soils. In general, the OTUs that were more abundant in the MEDIUM soil were more closely related with known genera and species, for example, Halosarcina palida, Halorhabdus tiamatea, Natronococcus amylolyticus, while OTUs more abundant in 157.2 dS/m soil clustered in different mostly unknown branches. [file 646820.f1.zip › 646820.f1/646820.f1.pdf]

Fig. S1

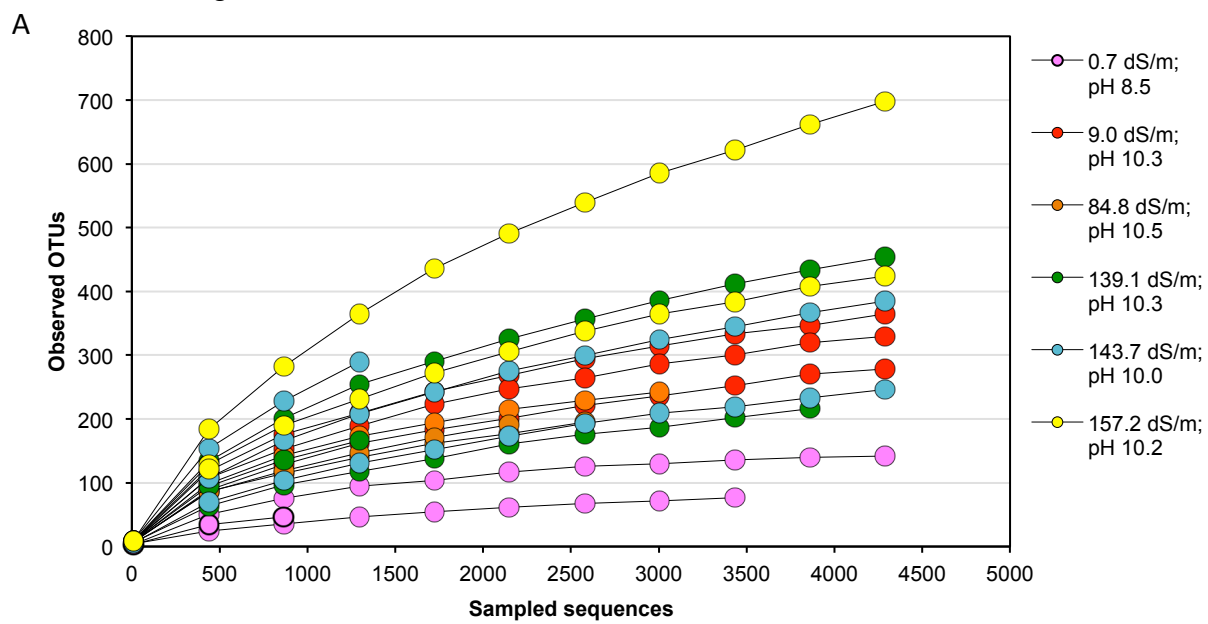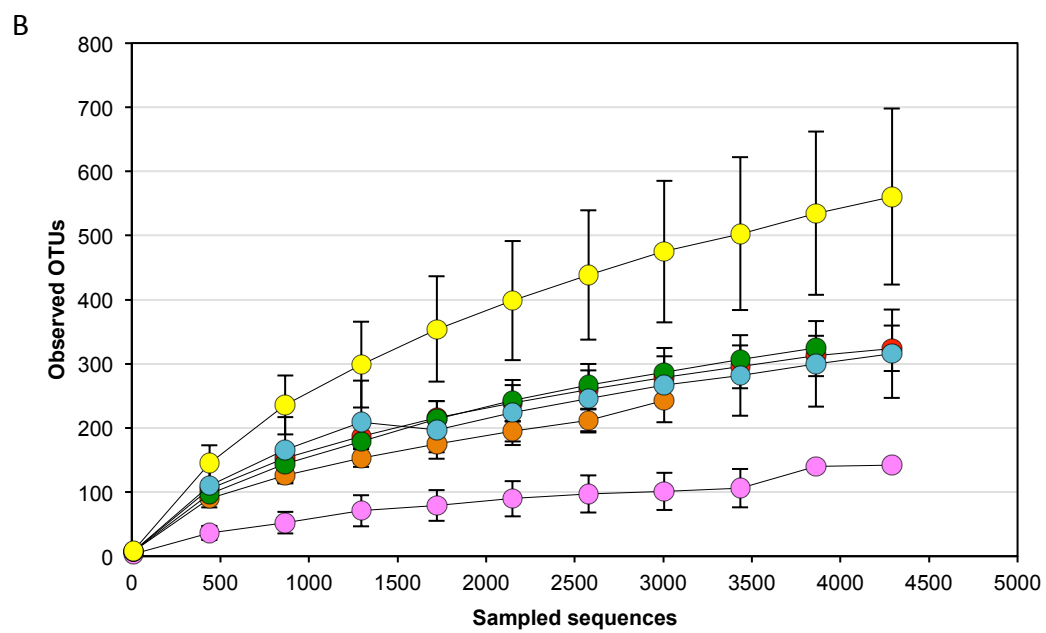

Fig. S2

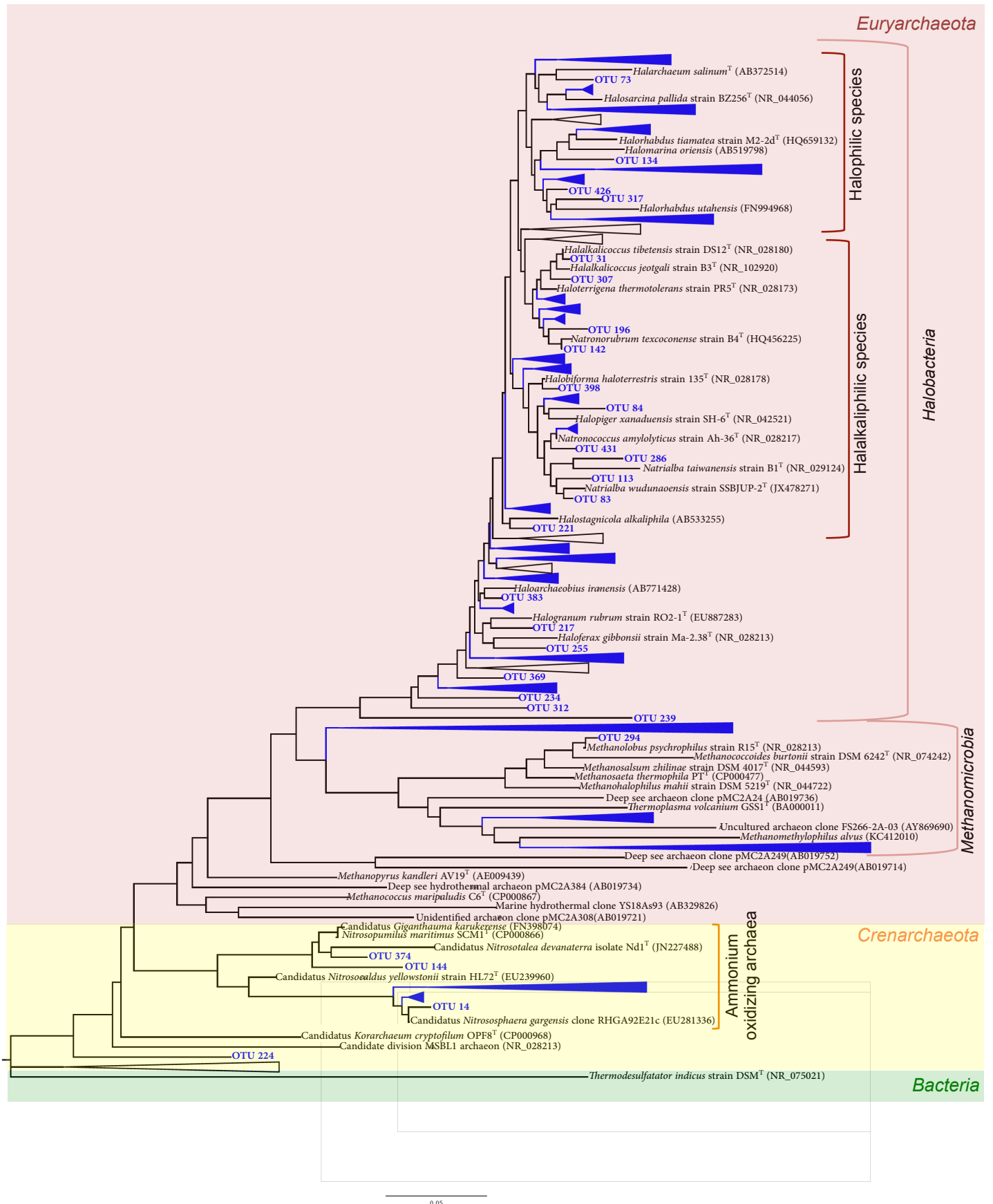

Supplement: Supplementary file 1 — Supplementary Figure 1: Rarefaction curves were constructed with the observed OTUs to verify the sampling–sequencing effort (Fig. S1). The observed species richness is larger in soil with electrolytic conductivity (EC) 157.2 dS/m, and lower in soil with EC 0.7 dS/m. Supplementary Figure 2: A phylogenetic tree was constructed with the sequences found in this study so as to have a more detailed phylogenetic placement of the archaeal phylotypes (Fig. S2). In the Crenarchaeota phylum, the mesophilic Crenarchaeota (ammonia oxidation clade) grouped the majority of the OTUs from the 0.7 dS/m soil. A group of abundant OTUs in this soil was closely affiliated with Candidatus Nitrososphaera gargensis recently suggested as the phylum Thaumarchaeota. OTU 144 with a high abundance in the 0.7 dS/m and 9.0 dS/m soils represented a deep branch in the group 1.1b or soil Crenarchaeota. Some OTUs belonging to Halobacteriales were detected in both the 9.0 and 139.1 dS/m soils. In general, the OTUs that were more abundant in the MEDIUM soil were more closely related with known genera and species, for example, Halosarcina palida, Halorhabdus tiamatea, Natronococcus amylolyticus, while OTUs more abundant in 157.2 dS/m soil clustered in different mostly unknown branches. [file 646820.f1.zip › 646820.f1/646820.f2.pdf]
